# Evaluation of KRAS^G12C^ inhibitor responses in novel murine KRAS^G12C^ lung cancer cell line models

**DOI:** 10.3389/fonc.2023.1094123

**Published:** 2023-02-08

**Authors:** Daniel J. Sisler, Trista K. Hinz, Anh T. Le, Emily K. Kleczko, Raphael A. Nemenoff, Lynn E. Heasley

**Affiliations:** ^1^ Department of Craniofacial Biology, University of Colorado Anschutz Medical Campus, Aurora, CO, United States; ^2^ Eastern Colorado VA Healthcare System, Rocky Mountain Regional VA Medical Center, Aurora, CO, United States; ^3^ Department of Medicine, University of Colorado Anschutz Medical Campus, Aurora, CO, United States

**Keywords:** KRAS, MRTX-1257, AMG-510, lung cancer, orthotopic model

## Abstract

**Introduction:**

The KRAS(G12C) mutation is the most common genetic mutation in North American lung adenocarcinoma patients. Recently, direct inhibitors of the KRAS^G12C^ protein have been developed and demonstrate clinical response rates of 37-43%. Importantly, these agents fail to generate durable therapeutic responses with median progression-free survival of ~6.5 months.

**Methods:**

To provide models for further preclinical improvement of these inhibitors, we generated three novel murine KRAS^G12C^-driven lung cancer cell lines. The co-occurring NRAS^Q61L^ mutation in KRAS^G12C^-positive LLC cells was deleted and the KRAS^G12V^ allele in CMT167 cells was edited to KRAS^G12C^ with CRISPR/Cas9 methods. Also, a novel murine KRAS^G12C^ line, mKRC.1, was established from a tumor generated in a genetically-engineered mouse model.

**Results:**

The three lines exhibit similar *in vitro* sensitivities to KRAS^G12C^ inhibitors (MRTX-1257, MRTX-849, AMG-510), but distinct *in vivo* responses to MRTX-849 ranging from progressive growth with orthotopic LLC-NRAS KO tumors to modest shrinkage with mKRC.1 tumors. All three cell lines exhibited synergistic *in vitro* growth inhibition with combinations of MRTX-1257 and the SHP2/PTPN11 inhibitor, RMC-4550. Moreover, treatment with a MRTX-849/RMC-4550 combination yielded transient tumor shrinkage in orthotopic LLC-NRAS KO tumors propagated in syngeneic mice and durable shrinkage of mKRC.1 tumors. Notably, single-agent MRTX-849 activity in mKRC.1 tumors and the combination response in LLC-NRAS KO tumors was lost when the experiments were performed in athymic *nu/nu* mice, supporting a growing literature demonstrating a role for adaptive immunity in the response to this class of drugs.

**Discussion:**

These new models of murine KRAS^G12C^ mutant lung cancer should prove valuable for identifying improved therapeutic combination strategies with KRAS^G12C^ inhibitors.

## Introduction

Lung cancer is the second most common cancer diagnosed in men and women, and accounts for the highest proportion of cancer-related deaths (1). In 2022, an estimated 240,000 patients will be diagnosed with lung cancer, resulting in an estimated 130,000 deaths (1). Lung adenocarcinomas (LUAD) account for ~ 30-40% of lung cancer patients and are clinically defined by their oncogenic driving mutations (2). Recent advances in precision medicine have led to the clinical approval of small molecule inhibitors that directly target oncogenic forms of EGFR, ALK, ROS1, RET, NTRK and BRAF mutant cancers. While pharmacological targeting of oncogenic receptor tyrosine kinases (RTKs) has experienced great success, other dominant oncogenic drivers including KRAS could not be directly targeted until recently. Mutations in the KRAS oncogene occur in ~30% of LUAD and inhibitors targeting the most common mutational subset of this gene (glycine to cysteine at codon 12) have been developed and gained FDA approval for use in a second line setting (3, 4). Early clinical data demonstrate that the KRAS^G12C^ inhibitor sotorasib (AMG-510) achieved an objective response rate of 37.1% and the median duration of response was 11.1 months with a comparatively short progression free survival of 6.8 months (5). These data indicate that single agent KRAS^G12C^ inhibitor activity is short-lived and as previous research has shown, ERK reactivation through both bypass signaling and acquired resistance mechanisms may drive these abbreviated responses (6, 7). These findings argue for the development of rational therapeutic combinations that may prolong ERK inhibition in a targeted manner to bolster duration of response.

KRAS^G12C^ inhibitors function to covalently bind KRAS^G12C^ in its GDP-bound (inactive) state. Distinct from other KRAS codon 12 alleles, KRAS^G12C^ retains intrinsic GTP hydrolysis capacity (8), highlighting proteins upstream of KRAS signaling as attractive candidates to target in combination with KRAS^G12C^ inhibitors (6). This upstream targeting helps prevent KRAS activation and nucleotide exchange for GTP, thereby making more GDP-bound KRAS available for KRAS^G12C^ inhibitor binding. The tyrosine-protein phosphatase SHP2/PTPN11 has emerged as a promising therapeutic target (9). This protein acts as a mediator associated with the downstream stimulation of the RAS/RAF/MEK/ERK pathway that promotes MAPK signal activation by dephosphorylating activating phospho-tyrosine residues within the RAS-GAPs, NF1 and p120RASGAP (10–12). SHP2 inhibition alone has little effect on reducing KRAS-driven tumor cell growth, but in combination with a MEK inhibitor resulted in synergistic tumor shrinkage (13). Another attractive aspect of targeting SHP2 derives from its functional repression of JAK/STAT and immune-related signaling pathways (14). Targeting this protein with allosteric SHP2 inhibitors promotes anti-tumor immunity, including enhancement of T cell cytotoxic function and immune-mediated tumor regression *via* a variety of different mechanisms (14). SHP2 has also been shown to abrogate KRAS^G12C^ inhibitor-specific responses in some models (15). Clinically, SHP2 inhibitors have exhibited encouraging disease control rates of 67% for advanced NSCLC patient with KRAS mutations, although significant adverse events related to toxicity were observed. Still, the ability to target both mutant KRAS signaling, as well as immune related signaling makes this combination appealing.

Our lab and others have shown the importance of innate and adaptive immunity in driving pre-clinical and clinical responses to oncogene-targeted therapies (16–23). Thus, comprehensive assessment of the therapeutic potential of oncogene-targeted agents requires immune competent murine models. To date, few murine models of KRAS^G12C^ lung cancer exist to test the effects of KRAS^G12C^ inhibitor in immune competent hosts. Herein, we have developed three new murine KRAS^G12C^-dependent lung cancer cell lines and tested their sensitivity to a KRAS^G12C^ inhibitor as a monotherapy and in combination with a SHP2 inhibitor *in vitro* as well as *in vivo* using an orthotopic model of lung tumor growth. The findings reveal superior activity of the drug combination in immune competent, but not immune-deficient mice.

## Materials and methods

### Cell culture

All human and murine cell lines were cultured in RPMI-1640 media (Corning, Tewksbury, MA) supplemented with 5% fetal bovine serum (FBS) and 1% penicillin–streptomycin (Sigma-Aldrich, St. Louis, MO) at 37°C in a humidified 5% CO_2_ incubator. All human cell lines used in this study were submitted to fingerprint analysis to confirm authenticity within a year of performing the studies described herein. A reference STR genotype was acquired through IDEXX Bioresearch for all murine lines and was later used to confirm genotypic integrity. CMT167 cells (24) and Lewis Lung Carcinoma cells (LLC) were obtained from the lab of Dr. Raphael Nemenoff. All cell lines were periodically tested for mycoplasma infection. To avoid cross-contamination and phenotypic changes, cells were maintained as frozen stocks and cultured for only two to four weeks before use in experiments. Authentication of cell lines based on morphology and growth curve analysis was performed regularly. No phenotypic changes were observed through the duration of the study.

### KRAS^G12C^ mouse tumor cell line generation

B6;129S4-*Kras^em1Ldow^*/J mice (Lukas Dow, Jackson Labs stock #033068) were crossed with B6.129P2-*Trp53^tm1Brn^*/J mice (Jackson Labs stock #008462). F1 hybrid mice were genotyped for KRAS and p53 status using PCR on genomic DNA and the following primer pairs: KRAS^G12C^, LSL-Kras Common 5’-TCCAATTCAGTGACTACAGATG-3’, LSL-Kras Mutant 5’-CTAGCCACCATGGCTTGAGT-3’, LSL-Kras Wildtype 5’-ATGTCTTTCCCCAGCACAGT-3’); Trp53, FWD 5’-GGTTAAACCCAGCTTGACCA-3’, REV-5’-GGAGGCAGAGACAGTTGGAG-3’). All PCR reactions employed PCRBIO VeriFi™ mix (PCRBIO, Wayne, Pennsylvania) according to manufacturer’s protocol with 60°C annealing temperature. Mice heterozygous for KRAS^G12C^ and varying p53 backgrounds (p53^+/+^, p53^+/flox^, and p53^flox/flox^) were inoculated with Ad-Cre intratracheally and submitted to microCT imaging once monthly until tumor burden was detected. Tumors were harvested, minced, plated on tissue culture plastic and cultured until the stable cell line, mKRC.1, was obtained.

### CRISPR-Cas9 genome editing

#### NRAS knockout

A crRNA was designed targeting exon 5 of genomic mouse NRAS using IDT (IDT, Iowa, City, IA) online software (5’-CACGAACTGGCCAAGAGTTA-3’). LLC cells (30,000) were plated in a 35 mm^2^ plate and cultured for 16 hrs. The NRAS targeting crRNA, ATTO550-labelled tracrRNA and recombinant Cas9 were assembled into ribonucleoprotein (RNP) particles according to IDT manufacturer’s protocol. Assembled RNPs were packaged using Lipofectamine RNAiMAX transfection reagent (ThermoFischer Scientific) according to manufacturer’s instructions, and transfection mixture was added to LLC cells. After a 24-hour incubation, cells were trypsinized, rinsed with 5 mL of PBS, and resuspended into a single-cell suspension in 1 mL of PBS. Single cells were flow sorted into 96-well plates using the MoFlo XDP100 cell sorter for ATTO550 positivity (Beckman Coulter) (University of Colorado Anschutz Medical Campus Cancer Center Flow Cytometry Core). For proper compensation of flow cytometry channels, unstained cell lines were used. Single cell clones were expanded, genomic DNA collected, and assessed for NRAS perturbations *via* PCR and Sanger Sequencing. Two independent clones were identified and indicated as LLC 23 NRAS KO and LLC 46 NRAS KO.

#### KRAS G12V to G12C

A crRNA targeting exon 1 of genomic mouse KRAS (5’-GACTGAGTATAAACTTGTGG-3’) using IDT online software was designed in addition to single stranded donor oligonucleotides (ssODN) homology-directed repair templates for both the positive and negative strands, the sequence for which is: 5’-ATGACTGAGTATAAACTTGTCGTCGTTGGAGCTTGCGGCGTAGGCAAGAGCGCCTTGACGATACAGCTAATTCAGA-3’. CMT167 KRAS^G12C^ cell lines were generated by the same protocol used for generating LLC NRAS knockout cell lines with the addition of two steps. 1. Prior to transfection, CMT167 cells were incubated with 30 μM HDR Enhancer (IDT) for 1 hour. 2. ssODN’s were added to the transfection mixture and co-transfected into cells along with assembled RNP’s. Single cell subclones from flow sorting were expanded and analyzed for endogenous KRAS^G12C^ mutations.

### RNA-seq and bioinformatic analysis

RNA was submitted to the University of Colorado Sequencing Core where library preparations were generated, and RNA was sequenced on the NovaSeq 6000 to generate 2 x 151 reads. Fastq files were quality checked with FastQC, illumina adapters trimmed with bbduk, and mapped to the mouse mm10 genome with STAR aligner. Counts were generated by STAR’s internal counter and reads were normalized to counts per million (CPM) using the edgeR R package. Differential expression was calculated using the limma R package and the voom function. Heatmaps were generated in Prism 9 (GraphPad Prism Software, San Diego, CA). Integrated Genomics Viewer analysis was completed by stripping section of sequence reads from RNAseq dataset and viewing in IGV software (25).

### Variant calling

Fastq files were quality checked with FastQC, illumina adapters trimmed with bbduk, and paired reads were mapped to the UCSC mm10 BWA genome with samtools bwa-mem. Output sam files were converted to bam format, sorted, and duplicates were marked with the picard tool, MarkDuplicates. Samtools “mpileup” and bcftools “call” multiallelic caller were used to call variants and generate variant call files (VCF). To identify variants unique to each cell line, bcftools “isec” was used on each cell lines VCF files filtered with bcftools “view” excluding “G/T = 0/0” and including variants with at least a read depth of 10 and a quality score of 20 compared to unfiltered parental VCF files. Variants private to each cell line (001.vcf) were then annotated with ANNOVAR mm10db including refGene, cytoBand, and genomicSuperDups. VCFs were filtered excluding identified “intronic”, and “intergenic” annotated variants under the “Func.refGene tab in addition to “synonymous”, “.”, and “other” under the “ExonicFunc.refGene” tab. Variants were confirmed by interrogating specific regions from the BAM files and uploading those reads to the integrated genomics viewer (IGV). A variant was considered detected reliably if the variant was present in > 10% of the total read depth at the variant position. All code and the parameters used are not included in this report but can be provided upon request.

### Cell proliferation assay

Cells were plated at 100 cells per well in 100 μL in 96-well tissue culture plates and allowed to attach for 24 hrs. Inhibitors were added at various doses as 2X concentrates in 100 μL. After incubation for 7-10 days, cell number per well was assessed using a CyQUANT Direct Cell Proliferation Assay (Invitrogen) according to the manufacturer’s instructions. The data are means and SEM (n=3) and presented as percent of the control values measured in DMSO-treated wells for each cell line.

### Enzyme linked immunosorbent assay (ELISA)

Conditioned media was collected from treated and untreated murine lung cancer cell lines. Chemokine levels were measured using the Invitrogen ELISA Kit (Quantikine mouse/human CXCL10/IP-10; Invitrogen, Carlsbad, CA) following manufacturer’s instructions. Absorbance was measured at 450 nm. The measured concentration in each sample was normalized to the total cellular protein per dish and the data are presented as pg/μg protein.

### *In Vivo* mouse studies

Eight-week-old female C57B/6J mice were purchased from Jackson Labs (Bar Harbor, ME) and eight-week-old *nu/nu* mice were purchased from Envigo (Indianapolis, IN). For tumor cell inoculation, parental LLC, LLC 46 NRAS KO, LLC 23 NRAS KO and mKRC.1 cells were grown to 75% confluence, harvested, and resuspended in sterilized phosphate buffered saline. Cells were counted and resuspended to a final concentration of 125,000 cells per 40uL injection. Cell suspensions were directly injected into the left lung of mice through the ribcage. Mice were randomized into groups to receive diluent control, RMC-4550 (30 mg/kg; MedChem Express), MRTX-849 (30 mg/kg; MedChem Express), or the combination by daily oral gavage until primary experimental endpoints were met. Treatment was initiated following confirmation that at least 75% of mice had calculatable primary left lung tumors *via* microCT imaging (CUD Small Animal Imaging Core). MicroCT imaging was conducted weekly and ITK-SNAP software (26) was used to calculate cross-sectional tumor volumes in cubic millimeters. Mice exhibiting signs of morbidity according to the guidelines set by the Institutional Animal Care and Use Committee (IACUC) were sacrificed immediately.

### Quantification and statistical analysis

Statistical Analysis: Prism 9 (GraphPad Software, San Diego, CA) was used to perform specific statistical analyses noted in the figure legends. Data are presented as the mean and standard error of the mean (SEM) as indicated. To calculate IC_50_ values, the dose-response data were submitted to the “log(inhibitor) vs. normalized response” function with the Prism 9 software. An unpaired Student’s t test (two-tail) was used to determine statistical significance, unless otherwise noted. The P values are denoted by *(P < 0.05), **(P < 0.01), ***(P < 0.001), and ****(P < 0.0001) and were corrected for multiple comparisons (Dunnett). Drug synergy with combinations of KRAS-G12C inhibitors and SHP2 inhibitors was determined with Combenefit, a free software tool for visualization, analysis and quantification of drug combination effects. Data from drug combination assays was processed using the HSA synergy model.

## Results

### Generation of murine lung cancer cell lines bearing KRAS^G12C^

The literature supports a role for innate and adaptive immunity in the overall tumoral responses to oncogene-specific inhibitors (16, 22, 27, 28). Herein, we generated three novel murine KRAS^G12C^-driven LUAD cell lines to permit investigation of KRAS^G12C^ inhibitor responses in immune-competent hosts.

#### LLC-NRAS-Q61L KO

Lewis lung carcinoma (LLC) are derived from a spontaneous lung tumor that arose in a C57BL/6 mouse (29) and has been investigated by our group for sensitivity of orthotopic lung tumors to PD1/PD-L1 axis inhibitors (30–33). In addition to a Trp53 missense mutation (R334P), Molina-Arcas et al. previously reported that a cell line derived from the LLCs (3LL) contained an NRAS-Q61H gain-of-function mutation in addition to a KRAS^G12C^ mutation (34). Variant calling analysis (See Materials and Methods) of our published RNAseq data (30) verified that our LLC cell line isolate also expressed NRAS-Q61L at the mRNA level (**Supplementary Figure S1A**). To render this cell line fully dependent on KRAS^G12C^, a guide RNA (gRNA) targeting exon 5 of the murine NRAS gene was introduced into parental LLC as a complex with Cas9 and single-cell transfectants were isolated and screened for functional NRAS knockout. Genetic NRAS knockout (KO) in 2 independent LLC clones (LLC 23 NRAS KO and LLC 46 NRAS KO) was validated by RNAseq showing a large deletion near the gRNA targeting region of exon 5 of NRAS (**Supplementary Figure S1B**).

#### CMT-167 KRAS-G12V to G12C

Like LLC cells, the CMT-167 cell line was isolated from a spontaneous lung tumor arising in a C57BL/6 mouse (24) and bears an oncogenic KRAS^G12V^ mutation. CMT167 cells express wild-type Trp53. CRISPR-Cas9-mediated, homology-directed repair was deployed to edit the endogenous G12V mutation to a G12C mutation. Ribonucleoprotein (RNP) particles containing Cas9, a single-stranded oligonucleotide DNA template and a gRNA targeting a sequence within exon 2 of murine KRAS were prepared and transfected into parental CMT cells. Following single cell flow sorting, clones were submitted to functional screening assessed for acquired sensitivity to KRAS^G12C^ inhibitors. Putative positive clones that successfully underwent homology-directed repair to contain the KRAS^G12C^ mutation were submitted to RNAseq and expression of the G12C allele was verified. RNA sequencing and IGV analysis confirmed an indel in one allele of murine KRAS and a successful recombination containing both KRAS^G12C^ as well as the predicted wobble position switches engineered to prevent Cas9 re-cutting of successfully recombined KRAS^G12C^ gene (**Supplementary Figure S1C**).

#### Novel KRAS^G12C^ cell line from KRAS-G12C GEMM

KRAS^LSL-G12C/+^ mice developed by the Dow lab (35) were obtained from Jackson Labs (B6;129S4-Kras^em1Ldow^/J) and crossed with Trp53^fl/fl^ mice (from Jackson Labs; B6.129P2-Trp53^tm1Brn^/J). The resulting KRAS^G12C/+^; Trp53^fl/fl^ mice were identified by genotyping and submitted to intratracheal administration of Ad-Cre as previously described (36) (**Supplementary Figure S2A**). Approximately 29 weeks post Adeno-Cre virus (Ad-Cre) administration, dispersed and invasive solid lung tumors were identified in the dissected lungs of a mouse. The tumors were dissociated into single cells and passaged *in vitro* to yield the murine KRAS-G12C positive, Trp53 null cell line, mKRC.1 (**Supplementary Figures S2A, B**).

Variant calling of RNAseq data revealed 8 and 23 novel insertion/deletion mutations in LLC 23 NRAS KO and LLC 46 NRAS KO, respectively, that were not detected in any of the RNAseq reads from parental LLC cells (**Supplemental Table S1**). Analysis of the CRISPR/Cas9-edited CMT-167 lines revealed 25 and 36 novel insertion/deletion mutations in CMT KRAS-G12C.54.10 and CMT KRAS-G12C.55, respectively that were not detected in any of the RNAseq reads from parental CMT-167 cells (**Supplementary Table S1**). None of the indels reside in genes with reported functions in MAPK pathway signaling.

The LLC NRAS KO and CMT KRAS-G12C cell lines were submitted to *in vitro* proliferation assays to assess if the genetic perturbations altered baseline cell growth relative to the parental lines. A minimal effect of NRAS-Q61L knockout in LLC cells was observed on baseline *in vitro* proliferation rates (**Supplementary Figure S3A**). By contrast, the rates of tumor growth from orthotopically implanted LLC 46 NRAS KO and LLC 23 NRAS KO cells were markedly reduced relative to parental LLC cells (**Supplementary Figure S3B**). Thus, the NRAS-Q61L significantly contributes to *in vivo* transformed growth of LLC cells. The edited CMT-KRAS-G12C cells exhibited significantly reduced *in vitro* proliferative rates compared to parental CMT-KRAS-G12V cells (**Supplementary Figure S3C**) and is consistent with previous findings that KRAS^G12V^ has lower intrinsic GTP hydrolysis (8) and cell lines expressing G12V mutations form more aggressive tumors than G12D or G12C mutations (37). In addition, the CRISPR/Cas9-mediated editing of the CMT cells resulted in deletion of the unedited KRAS-G12V allele so that both clones bear a single copy of KRAS-G12C. Thus, reduced copy number of the oncogenic KRAS allele may contribute to the reduced basal growth rate. Pilot experiments with orthotopic inoculation of CMT-KRAS-G12C cells in syngeneic C57BL/6 mice revealed formation of small primary tumors within the left lung lobe, but extensive growth in pleural and epicardial sites that required early euthanasia of the mice. As a result, *in vivo* experiments with the CMT KRAS-G12C cell lines were not further pursued in this study.

### Sensitivity of novel KRAS^G12C^ murine cell lines to KRAS^G12C^ and MEK inhibitors

**Figure 1:**
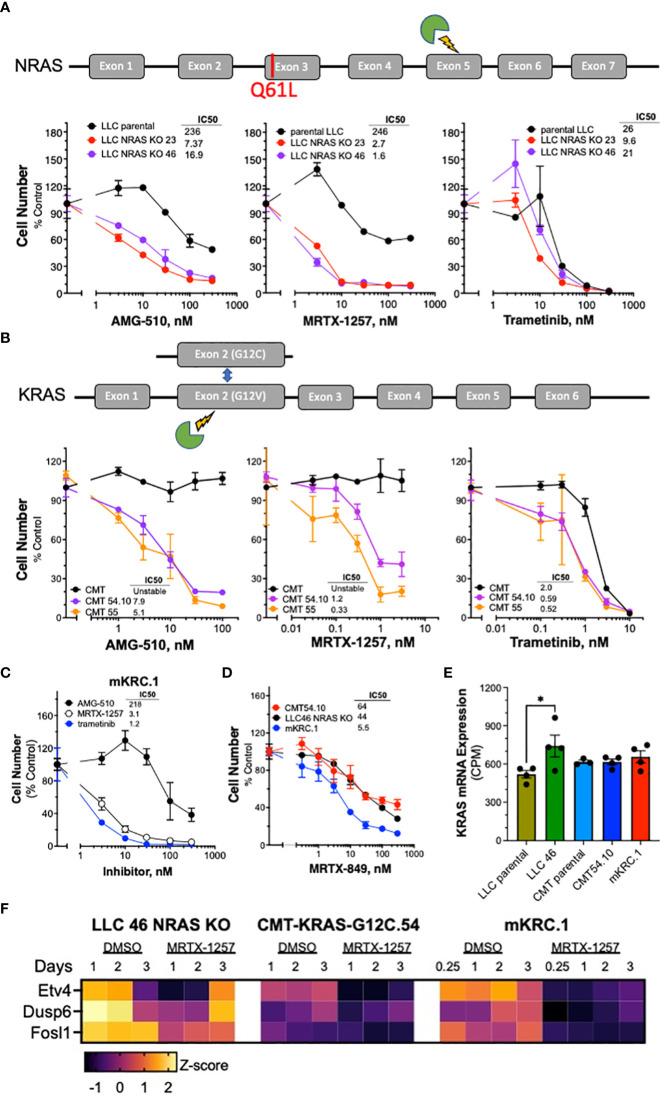
Murine KRAS^G12C^-mutant lung cancer cell lines demonstrate sensitivity to KRAS^G12C^ and MEK inhibitors. Schematic of CRISPR/Cas9 strategies are overlayed above each respective cell line panel. **(A)** Parental LLCs and 2 independent LLC clones harboring NRAS KO, **(B)** CMTs and 2 independent CMT clones with KRAS G12C substitutions. The parental and edited murine cell lines as well as **(C)** mKRC.1 were plated at 100-200 cells/well in triplicate in a 96-well plate, and then treated with increasing concentrations of the KRAS^G12C^ inhibitors MRTX-1257, AMG-510, MRTX-849 **(D)** and the MEK inhibitor, trametinib. Cell number was assessed 7-10 days later with the CyQuant assay. The data are the means ± SEM of triplicate determinations and presented as percent of the values measured in DMSO control wells. The experiments shown are representative of 2 to 3 independent experiments. The IC_50_ values were calculated with Prism 9 and the “log(inhibitor) vs. normalized response” function and presented within the individual dose-response curves as shown. Also, the IC_50_ values and their associated 95% confidence intervals are presented in **Supplementary Table S2**. **(E)** RNAseq data were used to compare the levels of KRAS mRNA (in CPMs) in the indicated murine KRASG12C-positive cell lines. The values represent the mean and SEM of 3-4 independent values. The data were analyzed by one-way ANOVA and only the increased KRAS mRNA levels in LLC 46 NRAS KO cells was significantly different from the other cell lines. **(F)** LLC 46 NRAS KO, CMT-KRAS-G12C.54 and mKRC.1 cells were treated for 6 hrs to 3 days with DMSO or 30 nM MRTX-1257, total RNA was collected and submitted to RNAseq. The expression levels (in CPMs) of the indicated MAP kinase pathway target genes were converted to Z-scores and presented as a heatmap.

The sensitivity of the murine KRAS^G12C^ lung cancer cell lines to the KRAS^G12C^ inhibitors MRTX-1257 and AMG-510 as well as the MEK1/2 inhibitor, trametinib was assessed with *in vitro* growth assays (see Materials and Methods). Knockout of NRAS increased the sensitivity of LLC NRAS KO cell lines to both KRAS^G12C^ inhibitors (AMG-510, 14-34 fold; MRTX-1257, 82-148 fold) compared to parental LLC cells (**Figure 1A**), further supporting that mutated NRAS-Q61H significantly contributes as an oncogenic driver in these cells. This finding is consistent with that reported previously in the 3LL derivative of LLC cells (34). LLC NRAS KO cell lines exhibited similar sensitivity to trametinib as parental LLCs (**Figure 1A**), indicating that NRAS-Q61L serves as an additional proximal driver of MAPK signaling in LLC cells. Compared to parental CMT cells which are completely insensitive to MRTX-1257 and AMG-510 at concentrations of 3 nM and 300 nM, respectively, the CMT KRAS^G12C^-engineered cell lines were highly sensitive to these agents with IC_50_ values in the low nanomolar range (**Figure 1B**). Interestingly, parental CMT cells bearing KRAS^G12V^ were less sensitive to trametinib than the CMT KRAS^G12C^ clones (**Figure 1B**). This finding may also reflect the disruption of the non-edited KRAS-G12V allele as a result of the CRISPR editing resulting in a single functional KRAS-G12C gene in the CMT-54 and CMT-55 clones. The reduced KRAS copy number may also contribute to their decreased proliferation rates observed *in vitro* (**Suppl. Figure S3C**). Finally, the mKRC.1 cell line exhibits sensitivity to MRTX-1257 and AMG-510 (**Figure 1C**). RNAseq data from the parental and engineered cell lines was interrogated for KRAS mRNA levels. LLC 46 NRAS KO cells exhibited 1.4-fold higher KRAS mRNA levels relative to parental LLC cells (p = 0.04, one-way ANOVA), but no other significant differences in KRAS mRNA expression were noted (**Figure 1E**). In addition to cell growth, mRNA expression levels of the MAP kinase pathway target genes (38, 39) ETV4, DUSP6 and FOSL1 measured by RNAseq were markedly reduced in LLC 46 NRAS KO, CMT-KRAS-G12C.54.10 and mKRC.1 cells after 1 to 2 days of MRTX-1257 treatment (**Figure 1F**). Evidence for pathway re-activation was evident by 3 days of MRT-1257 treatment, especially in LLC 46 NRAS KO cells.

Analysis of MRTX-1257 and AMG-510-sensitivity in a panel of 13 human KRAS^G12C^-mutant lung cancer cell lines revealed IC_50_ values for these two drugs that ranged from 0.1 to 356 nM for MRTX-1257 and 0.3 to 2534 nM for AMG-510 (**Supplementary Figures S4A, B**). The inhibitor sensitivity exhibited by the murine KRAS^G12C^ cell lines (**Figure 1**) overlapped with the most sensitive human lung cancer cell lines bearing KRAS^G12C^, indicating that co-occurring mutations present in many of the human lines markedly reduce their sensitivity to MRTX-1257 and AMG-510 similar to the activity of GTPase-deficient NRAS in parental LLC cells. Overall, the murine LUAD cell lines exhibit sensitivity to KRAS^G12C^ inhibitors that are consistent with human KRAS^G12C^ mutant cell lines.

### SHP2 inhibition synergizes with KRAS^G12C^ inhibitors *in vitro* leading to enhanced chemokine response

In a pilot study, LLC 46 NRAS KO cells were propagated as orthotopic tumors in the left lung of C57BL/6 mice and treated with diluent or the MRTX-1257 clinical-grade analogue, MRTX-849 (30 mg/kg daily). The results revealed a modest reduction in the rate of growth by single agent KRAS^G12C^ inhibitor (data not shown) and indicate that combination therapy with MRTX-849 will be required to achieve clinically significant anti-tumor responses. In this regard, the protein tyrosine phosphatase, PTPN11/SHP2, has emerged as a promising upstream target as it functions to dephosphorylate multiple proteins leading to sustained KRAS GTP cycling and activation (11–13, 40, 41), and highly specific inhibitors are available. SHP2 also plays a role in negatively regulating JAK-STAT signaling through dephosphorylation of activated STAT proteins (42) and T cell receptor (TCR) signaling within lymphocytes resulting in decreased T cell effector function (14, 15). Both JAK-STAT signaling, and adaptive immunity and T cell function have been shown to be necessary for oncogene-targeted therapy response (14–17).

**Figure 2:**
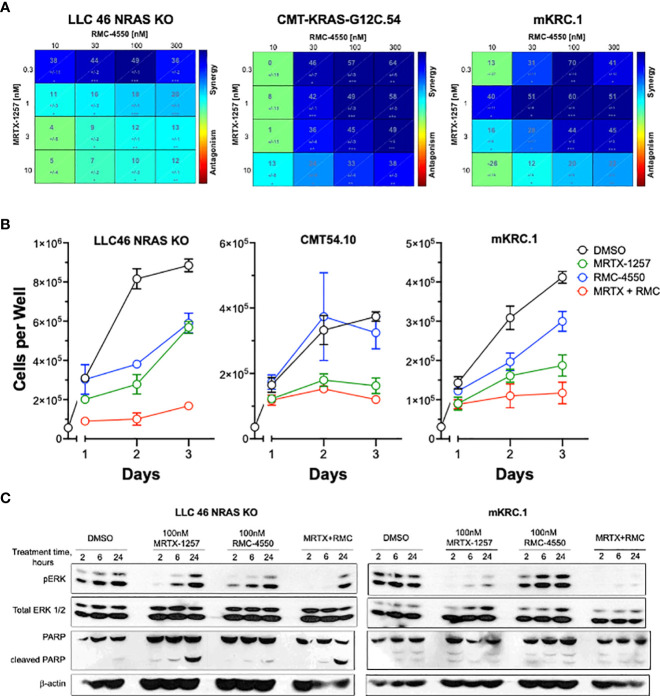
SHP2 inhibitor synergizes with KRAS^G12C^ inhibitor in KRAS-G12C-driven NSCLC cell lines. **(A)** Murine cell lines driven by KRAS^G12C^ were plated at 100 cells per well in a 96 well plate and treated with increasing concentrations of either MRTX-1257, RMC-4550, or in combination for 7-10 days, and then assessed for cell growth *via* CyQuant cell growth assays. HSA synergy was calculated using Combenefit software. **(B)** LLC 46 NRAS KO, CMT-KRAS-G12C.54 and mKRC.1 cells were seeded in 24-well plates and treated with DMSO diluent, 10 nM MRTX-1257, 300 nM RMC-4550 or the combination. Cell numbers were directly counted on days 1-3 following drug addition. The data are the means and SEM of triplicates. **(C)** LLC 46 NRAS KO and mKRC.1 cells were plated and treated with DMSO control, MRTX-1257 (100 nM), RMC-4550 (100 nM), or the combination for 2, 6, and 24 hours. Cell lysates were collected and immunoblotted for phospho-ERK, total ERK1/2 protein, PARP and β-actin.

To test the effect of the SHP2 inhibitor RMC-4550 in combination with KRAS^G12C^ inhibition, we plated LLC 46 NRAS KO, CMT-KRAS-G12C.54 and mKRC.1 cell lines in 96-well plates and assessed cell growth in response to a range of concentrations of MRTX-1257 and RMC-4550 alone and in combination. Analysis of the resulting data (see **Suppl. Figure S5** for primary data) with Combenefit (43) revealed strong *in vitro* synergy with combinations of MRTX-1257 and RMC-4550 in these cell lines (**Figure 2A**). Two human KRAS-G12C-positive lung cancer cell lines, Calu1 and H2030, were similarly submitted to growth assays with combinations of MRTX-1257 and RMC-4550. Analysis of the data with Combenefit indicated synergistic drug interactions in these human cell line models as well (**Suppl. Figure S6**). Also, combination of these inhibitors yielded greater inhibition of pERK in mKRC.1 and LLC 46 NRAS KO over time compared to either single agent alone (**Figure 2C**), suggesting this combination induces synergistic growth inhibition, in part, through prevention of ERK reactivation. To assess if the MRTX-1257/RMC-4550 combination exerts cytostatic or cytotoxic effects, LLC 46 NRAS KO, CMT-KRAS-G12C.54 and mKRC.1 cells were seeded in 24-well plates and treated with DMSO diluent, 10 nM MRTX-1257, 300 nM RMC-4550 or the combination. Cell numbers were directly counted and the results (**Figure 2B**) suggest that the drug combination induces a cytostatic, but not a cytotoxic effect in the three cell lines. Also, immunoblot analysis of full-length PARP and cleaved PARP, a biochemical measure of apoptosis, revealed modest induction of PARP cleavage in LLC46 cells with single-agent MRTX-1257, but not further increased by combining with RMC-4550 (**Figure 2C**). No PARP cleavage was noted in mKRC.1 cells treated with either drug alone or in combination.

### Therapeutic response to KRAS^G12C^ inhibitor, alone and in combination with SHP2 inhibitor in orthotopic *in vivo* models

**Figure 3:**
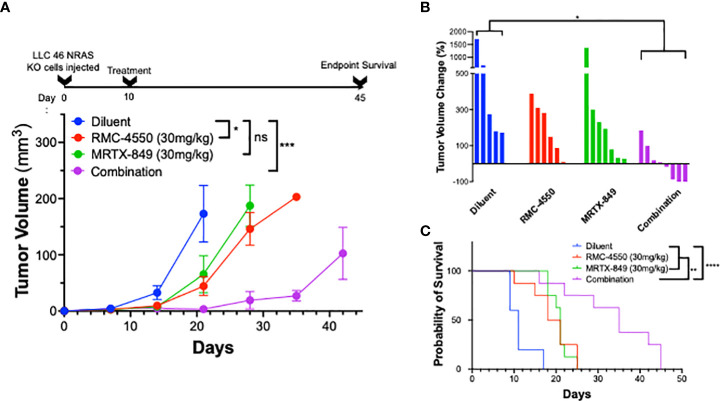
KRAS^G12C^ inhibitors in combination with SHP2 inhibitors exhibit improved efficacy compared to either treatment alone *in vivo*. LLC 46 NRAS KO cells were orthotopically implanted into the left lung of C57BL/6 mice and allowed to establish for 7 days before initial pre-treatment μCT imaging. **(A)** Scheme showing the timeline of the experiment overlayed on tumor volume growth curves of diluent (n=5), RMC-4550 (n=6), MRTX-849 (n=7), and combination-treated (n=8) mouse cohorts. The data were analyzed by 2-way ANOVA with Tukey’s multiple comparisons test. **(B)** Waterfall plot showing percent change in tumor volume from pretreatment (day 7) to day 21 (11 days of treatment). The data were analyzed by Kruskal-Wallis test with Dunn’s multiple comparisons test. **(C)** Survival curves comparing all 4 groups over the course of the study with one-way ANOVA and multiple comparisons. ns indicates not significant.

**Figure 4:**
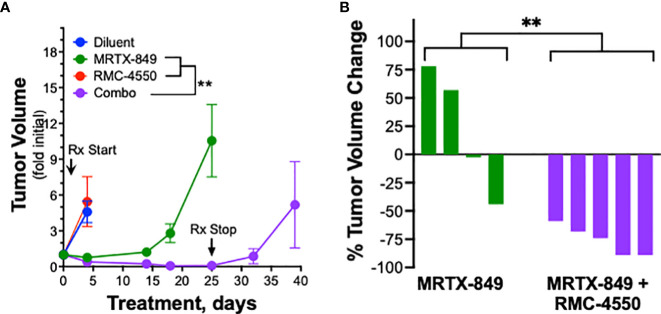
mKRC.1 tumors in C57BL/6 mice show single agent MRTX-849 response and synergistic tumor shrinkage in combination with RMC-4550. mKRC.1 cells were orthotopically implanted into the left lungs of C57BL/6 mice and allowed to establish for 14 days before initial pre-treatment μCT imaging. **(A)** Tumor volumes presented as fold of initial volumes (mean and SEM) from diluent, MRTX-849 (30mg/kg), RMC-4550 (30 mg/kg) and combination-treated mouse cohorts (n=4-5 per group) are shown. The arrows indicate days when treatment was initiated and terminated. The data were analyzed by 2-way ANOVA with Tukey’s multiple comparisons test. **(B)** Waterfall plot showing percent change in tumor volume (fold of initial) after 14 days of MRTX-849 or MRTX-849 plus RMC-4550 treatment. The data were analyzed by an un-paired t-test.

Based on the synergistic activity of the combination of MRTX-1257 and RMC-4550 *in vitro* (**Figure 2A**), the drug combination was tested in LLC 46 NRAS KO and mKRC.1 cells using an orthotopic lung tumor model (30). Following inoculation of LLC 46 NRAS KO cells into the left lung of C57BL/6 mice, tumors were assessed for growth over time using μCT imaging. Representative μCT images from this experiment are presented in **Supplemental Figure S7**. Treatment with single agent RMC-4550 or MRTX-849 resulted in marginally reduced rate of tumor growth compared to diluent control (**Figure 3A**). However, the combination of the two agents yielded initial tumor regression and suppressed tumor growth until tumors ultimately progressed despite continued treatment (**Figure 3A**). Assessment of the individual tumor responses at day 21 (11 days of treatment) among the experimental groups revealed no significant change in tumor volume with single agents compared to diluent control mice, although modest improvement in overall survival was observed (**Figures 3B, C**). By contrast, four of the eight mice treated with the combination of MRTX-849 and RMC-4550 experienced tumor shrinkage and significantly improved survival compared to diluent control and each single agent alone (**Figures 3B, C**). In contrast to the LLC 46 NRAS KO model, orthotopic lung tumors established with the mKRC.1 cell line exhibited a significant single-agent anti-tumor response to MRTX-849 (**Figure 4A** and representative μCT images in **Suppl. Figure S8**), although progression on continued drug treatment was observed within 18 days. RMC-4550 was ineffective as a single agent, but combination of MRTX-849 and RMC-4550 yielded durable tumor shrinkage to less than 10% of initial tumor volume. Assessment of individual tumor responses to 14 days of treatment with MRTX-849 alone or in combination with RMC-4550 is shown in **Figure 4B** and demonstrates shrinkage of all five of the combination-treated tumors relative to one of four in the MRTX-849 monotherapy group. When the daily MRTX-849 plus RMC-4550 treatment was terminated after 25 days, the residual tumor cells rapidly progressed over the subsequent 2 weeks, indicating that the combination treatment did not eliminate residual cancer cells in the mKRC.1 tumors.

### The MRTX-849 and/or RMC-4550 response is reduced in immune-deficient mice

**Figure 5:**
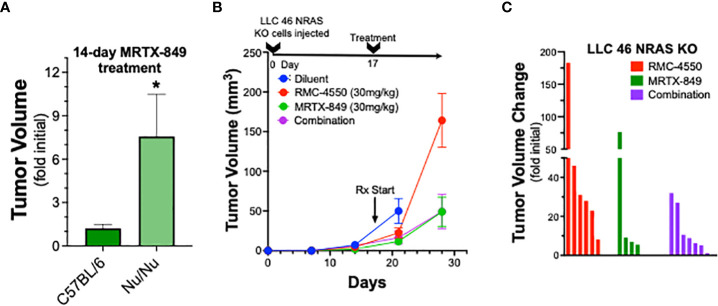
Therapeutic response of mKRC.1 and LLC 46 NRAS KO tumors to MRTX-849 and/or RMC-4550 in *nu/nu* mice. mKRC.1 and LLC 46 NRAS KO cells were orthotopically implanted into the left lung *of nu/nu* mice and allowed to establish for 7 days before initial pre-treatment μCT imaging. **(A)** Volumes (fold of initial; mean and SEM) of mKRC.1 tumors propagated in C57BL/6 (n=4) or *nu/nu* (n=3) mice treated for 14 days with MRTX-849 are shown. The data were analyzed by an unpaired t-test. **(B)** LLC 46 NRAS KO tumor volumes with time of treatment with diluent (n=8), MRTX-849 (n=10) or RMC-4550 (n=8) alone and in combination (n=8) are shown. **(C)** Waterfall plot showing changes in tumor volume after 11 days of treatment as fold of initial volumes. The data were analyzed by Kruskal-Wallis test with Dunn’s multiple comparisons test and no significant differences were detected.

To explore the role of adaptive immunity in contribution to the tumor response to KRAS^G12C^ inhibition alone or in combination with SHP2 inhibition, mKRC.1 cells were propagated as orthotopic tumors in the left lungs of athymic *nu/nu* mice. The findings in **Figure 5A** show that the MRTX-849 response (fold of initial volume after a 14-day treatment) in C57BL/6 and *nu/nu* hosts was 1.2 ± 0.3 and 7.6 ± 2.9, respectively. Similarly, LLC 46 NRAS KO cells were inoculated into the left lung of athymic *nu/nu* mice and tumors were allowed to establish. Subsequently, tumor-bearing *nu/nu* mice were treated with diluent control, RMC-4550, MRTX-849, or the combination. While either drug alone reduced tumor growth similar to the result in C57BL/6 mice (**Figure 3A**), the combination failed to yield any additional benefit (**Figure 5B**). This was further supported by assessing percent tumor volume change in the individual mice, as the combination treatment group was not different than the individual tumors within the single agent groups (**Figure 5C**). These results suggest a role for adaptive immune cells as participants in the MRTX-849 response in mKRC.1 tumors and the enhanced response to the MRTX-849 and RMC-4550 combination in LLC 46 NRAS KO tumors.

### Chemokine gene expression in KRAS^G12C^ cell lines and regulation by MRTX-1257 and RMC-4550

**Figure 6:**
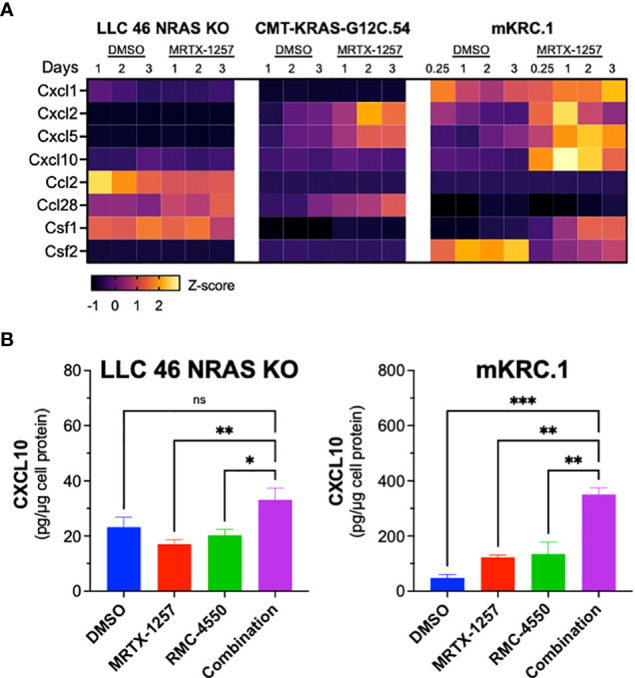
Chemokine mRNA and protein expression in MRTX-1257-treated murine KRAS^G12C^ cell lines. **(A)** LLC 46 NRAS KO, CMT-KRAS-G12C.54 and mKRC.1 cells were treated for 6 hrs to 3 days with DMSO or 30 nM MRTX-1257, total RNA was collected and submitted to RNAseq. The expression levels (in CPMs) of the indicated chemokine genes where normalized to Z-scores and graphed as a heatmap. **(B)** LLC 46 NRAS KO and mKRC.1 cells were plated in 6-well plates and treated for 2 days with either DMSO control, RMC-4550 (100 nM), MRTX-1257 (100 nM), or the combination. Conditioned cell media was collected and assessed for CXCL10 expression by ELISA. The data are presented as pg/μg of cellular protein and are the mean and SEM of 6 and 3 replicates for LLC 46 NRAS KO and mKRC.1, respectively. The data were submitted to 1-way ANOVA with multiple comparisons test. ns, not significant.

Previous studies have demonstrated that KRAS-SHP2-MAP kinase pathway-targeted agents induce expression of multiple chemokines in KRAS-mutant lung cancer cell lines (21, 44, 45) and may provide a mechanism by which oncogene-targeted drugs induce cross-talk between cancer cells and the tumor microenvironment. Interrogation of RNAseq data generated from LLC 46 NRAS KO, CMT-KRAS-G12C.54 and mKRC.1 cells treated *in vitro* for 6 hr to 3 days with 30 nM MRTX-1257 reveals distinct expression patterns of chemokines with defined anti- and pro-tumorigenic functions (46) (**Figure 6A**). LLC 46 NRAS KO cells constitutively expressed Ccl2 and Csf1 and MRTX-1257 modestly induced Ccl28 mRNA. By contrast, CMT-KRAS-G12C.54 cells exhibited low levels of the chemokines surveyed, but Cxcl2, Cxcl5 and Ccl28 mRNAs were induced upon MRTX-1257 treatment. Inspection of RNAseq data from mKRC.1 cells revealed constitutive expression of Cxcl1 as well as baseline expression of Csf2 that was reduced upon MRTX-1257 treatment. In addition, Cxcl2, Cxcl5, Cxcl10, and Csf1 were induced upon MRTX-1257 treatment. Among these secreted chemokines, Cxcl10 is defined as anti-tumorigenic through recruitment of T cells (47) and was strongly induced in mKRC.1 cells relative to LLC 46 NRAS KO and CMT-KRAS-G12C.54 cells. To confirm this enhanced expression in mKRC.1 cells, secreted levels of CXCL10 were measured by ELISA in conditioned medium obtained from LLC 46 NRAS KO and mKRC.1 cells treated for 2 days with DMSO, MRTX-1257, RMC-4550 and the combination of the two drugs. The combination of MRTX-1257 and RMC-4550 stimulated significant, but modest induction of CXCL10 protein compared to single agent MRTX-1257 or RMC-4550 in LLC 46 NRAS KO cells (**Figure 6B**). Moreover, mKRC.1 cells exhibited increased CXCL10 secretion to single agent MRTX-1257 or RMC-4550 and when combined, further increased levels of CXCL10 protein relative to the single agents (**Figure 6B**).

## Discussion

The murine KRAS^G12C^-driven lung cancer cell lines developed in this study provide novel models to investigate KRAS^G12C^ inhibitor responsiveness in the immune competent setting. This is critical since oncogene-targeted agents including KRAS^G12C^ inhibitors have been shown to initiate functional interactions with host immunity (27, 28, 34, 44, 45). The new lung cancer cell lines described herein uniformly exhibit high *in vitro* sensitivity to KRAS^G12C^ inhibitors and altered growth signaling in CMT KRAS^G12C^ clones compared to parental G12V cells. This is most likely due to varied downstream signaling dynamics among G12 mutations (8) as well as disruption of the unedited KRAS-G12V allele as a result of CRISPR-Cas9 editing yielding a single functional KRAS-G12C allele. Similar to observed clinical activity of single agent KRAS^G12C^ inhibitors in which overall response rates are lower than 45%, and the durability of treatment response is rather brief, our murine models replicate these differential responses through initial progressive disease (LLC NRAS KO) or rapid progression after initial shrinkage (mKRC.1) to MRTX-849 as a single agent. Thus, these new murine KRAS^G12C^-driven lung cancer cell lines propagated as orthotopic tumors reflect treatment responses in KRAS^G12C^-positive patients treated with single agent KRAS^G12C^ inhibitors (5, 48). The Downward group has reported several murine KRAS^G12C^-driven lung cancer cell lines including an NRAS-deleted derivative of the LLC model as well as KPAR^G12C^ and KPB6^G12C^ cells that exhibit varying degrees of *in vivo* sensitivity to KRAS^G12C^ inhibitors (28, 34, 44, 45). Similar to our findings with LLC 46 NRAS KO cells, their LLC derivative exhibited modest *in vivo* sensitivity to KRAS^G12C^ inhibitor monotherapy. Also, KPAR^G12C^, KPB6^G12C^ cells and CT26 cells of colorectal origin engineered to express the KRAS^G12C^ allele exhibited single agent KRAS^G12C^ inhibitor activity in syngeneic mice (27, 44, 45) similar to our findings with the mKRC.1 cell line. Two distinct KRAS^G12C^ GEMMs have been published (35, 49), although the mKRC.1 cell line described herein appears to be the sole stable cell line derived from a KRAS^G12C^ GEMM. In summary, the novel murine KRAS^G12C^ cell lines described in this study add to the growing collection of models to further explore experimental therapeutics involving KRAS^G12C^ inhibitors.

The studies with the LLC NRAS KO cells propagated orthotopically in C57BL/6 mice showed that combined MRTX-849 and SHP2 inhibitor, RMC-4550, induced tumor shrinkage, albeit transiently (**Figure 3**). Notably, this synergistic response in LLC 46 NRAS KO cells as well as the single-agent MRTX-849 response in mKRC.1 tumors was abrogated when the experiment was performed in athymic *nu/nu* mice (**Figure 5**), suggesting a contributing role from adaptive immunity. In this regard, Boumelha et al. demonstrated durable KRAS^G12C^ inhibitor responses in subcutaneous tumors with the KPAR^G12C^ model, but transient effects in Rag1^-/-^ mice. By contrast, the anti-tumor response in KPB6^G12C^ tumors was equivalent in syngeneic and immune-deficient hosts (44). In fact, a role for innate and adaptive immunity in contributing to anti-tumor responses to oncogene-targeted therapeutics is well-documented in the literature (16–19, 21–23, 27, 28). Our data show that KRAS^G12C^ inhibitors variably induce chemokines including CXCL10 capable of recruiting anti-tumorigenic immune cells such as NK, CD8 T, and dendritic cells into the TME, which is enhanced in combination with the SHP2 inhibitor (**Figure 6B**). Further, mKRC.1 cells more strongly induced CXCL10 upon *in vitro* MRTX-1257 and MRTX-1257/RMC-4550 combination treatment. A future goal would be to test the role of CXCL10 induction in the single agent MRTX-849 activity observed in mKRC.1 tumors compared to LLC NRAS KO cells. A testable hypothesis is that the extent of chemokine induction following KRAS G12C inhibition may contribute to the depth and durability of response. In addition to the previously noted studies demonstrating involvement of host immunity in therapeutic response, our own studies with murine EML4-ALK-driven (19) and EGFR-mutant (20) lung cancer cell lines demonstrate a contribution of adaptive immunity to durable responses to the ALK inhibitor, alectinib and the EGFR inhibitor, osimertinib, respectively. Current understanding surrounding the depth and duration of response to oncogene-targeted inhibitors suggests that the extent to which chemokine induction occurs with oncogene-specific therapies associates with the degree of response, as EGFR mutant lung cancer patients exhibiting greater interferon γ transcriptional responses to TKIs presented with longer progression-free survival (17). Identifying why some patients experience stronger immunogenic responses to oncogene-targeted inhibitors remains an unanswered question in the field and a deeper understanding may unveil novel mechanisms to increase objective responses and durability of treatment with oncogene-targeted agents in general.

Precision therapy with oncogene-targeted drugs has dramatically changed the outcomes for lung cancer patients with tumors positive for mutated EGFR or fusion kinases including ALK and ROS1 and others. In fact, TKIs are now FDA-approved first-line therapies for these subsets of lung cancer. By contrast, KRAS^G12C^ inhibitors remain second-line treatments for KRAS^G12C^-positive lung cancers due to lower objective response rates and shorter durations of treatment response relative to the clinical activity observed with anti-PD-1-based immunotherapy, either alone or in combination with cytotoxic chemotherapy (5). Completed clinical trials of sotorasib and adagrasib reveal response rates ranging from 37 to 43% and rather brief PFS (6 – 7 months) relative to that associated with TKIs and RTK-driven lung cancers (5). The lower efficacy of sotorasib and adagrasib as monotherapies supports their combination with other pathway-targeted agents or immune therapy for better anti-cancer activity. Inhibition of the PD-1/PD-L1 axis remains a primary treatment strategy for KRAS mutant lung cancer. Numerous studies have shown that the existence of an active IFN signature within the TME is required for response to immune checkpoint inhibitors (50, 51). Because oncogene-targeted inhibitors have been shown to induce an IFN signature within the TME (16, 17), numerous trials combining immune therapies with KRAS^G12C^ inhibitors have been initiated to see if KRAS inhibition can expand checkpoint inhibitor responses.

Among the targets being investigated for combined benefit with KRAS^G12C^ inhibitors, SHP2 inhibitors have gained interest due to their ability to impact both tumor cell autonomous MAPK signaling and JAK-STAT signaling, as well as regulation of T cell receptor signaling (6, 9, 14, 15, 21, 52, 53). Multiple SHP2 inhibitors are now in the clinical trial pipeline being tested as monotherapies, and in combination with KRAS^G12C^ inhibitors (53). In addition, emerging data supports the rationale of targeting upstream of KRAS GTP nucleotide cycling as KRAS^G12C^ inhibitors covalently bind KRAS in its GDP-bound (inactive) form (54). Unlike other codon 12 missense mutations, KRAS^G12C^ maintains most of its intrinsic GTP hydrolysis activity, allowing the KRAS protein to inactivate through GTP hydrolysis even in its mutant form (8). This represents an interesting therapeutic vulnerability as upstream blockade of KRAS signaling should impede KRAS GTP nucleotide cycling into its active form resulting in a higher ratio of KRAS-GDP, the substrate for KRAS^G12C^ inhibitors. In this regard, the SHP2 inhibitor RMC-4550 exhibits synergistic *in vitro* growth inhibition in combination with MRTX-1257 in all three cell lines tested. Moreover, RMC-4550 increases efficacy of MRTX-849 in the LLC NRAS KO orthotopic C57BL/6 model (**Figure 3A**), but not immune deficient hosts (**Figures 5B, C**), supporting an hypothesis that host immunity may contribute to this therapeutic effect. The pre-clinical KRAS^G12C^ models described in this report allow for deeper experimental exploration of the impacts of inhibiting SHP2 on both tumor cell autonomous signaling and non-autonomous targets such as T cell signaling. These models may also serve as pre-clinical models for other KRAS^G12C^ inhibitor combinations currently in the clinical trial pipeline such as inhibitors of EGFR and SOS (10).

## Data availability statement

The datasets presented in this study can be found in online repositories. The names of the repository/repositories and accession number(s) can be found below: https://www.ncbi.nlm.nih.gov/geo/,GSE207098.

## Ethics statement

The animal study was reviewed and approved by University of Colorado Institutional Animal Care and Use Committee.

## Author contributions

Conception and design: DS, TH, EK, RN, LH. Development of methodology: DS, TH, AL, RN, LH. Acquisition of data: DS, TH, LH. Analysis and interpretation of data: DS, TH, LH. Writing, review, and/or revision of the manuscript: DS, TH, EK, RN, LH. All authors contributed to the article and approved the submitted version.
