# Supplementary material for: Evaluation of KRASG12C inhibitor responses in novel murine KRASG12C lung cancer cell line models
Source: Front Oncol. 2023 Feb 8;13:1094123. doi: 10.3389/fonc.2023.1094123 (PMC9945252; doi:10.3389/fonc.2023.1094123)
Supplement: Supplementary file 1 [file DataSheet_1.pdf]

Supplementary Information for Evaluation of KRAS^{G12C} Inhibitor Responses in Novel Murine KRAS^{G12C} Lung Cancer Cell Line Models

Daniel J Sisler[1,3], Trista K Hinz[1,3], Anh T Le[2], Emily K Kleczko[2], Raphael A Nemenoff[2], Lynn E Heasley[1,3]*

Departments of [1]Craniofacial Biology and [2]Medicine, University of Colorado Anschutz Medical Campus, Aurora, CO

[3]Eastern Colorado VA Healthcare System, Rocky Mountain Regional VA Medical Center, Aurora, CO

***Corresponding Author:**

Lynn E. Heasley
Department of Craniofacial Biology
University of Colorado Anschutz Medical Campus
Aurora, Colorado
(303) 724-4578
Lynn.heasley@cuanschutz.edu

Running Title: Novel murine KRAS^{G12C}-dependent lung cancer cell lines

Supplemental Figures S1 – S8
Supplemental Table S1 and S2

A.

Chr	Genomic Frame	Gene	Mutation Function	Amino Acid Change	cytoBand
chr3	exonic	Nras	nonsynonymous SNV	Nras:NM_010937:p.Q61H	3qF2.2

B.

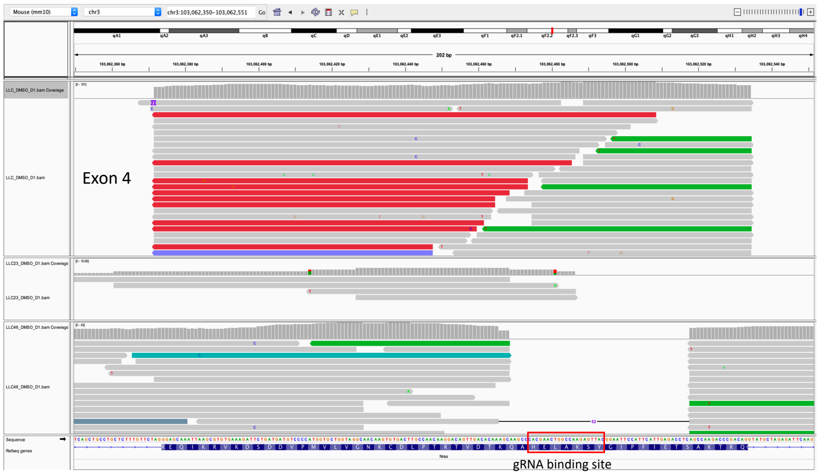

C.

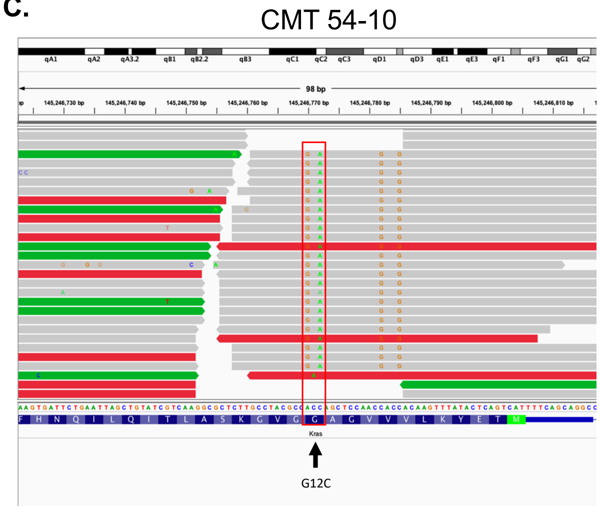

Supplemental Figure S1. Validation of NRAS Q61H mutation and CRISPR/Cas9 genomic editing. (**A**) Variant call analysis (See Materials and Methods) was performed on parental LLC RNA sequencing data. (**B**) Integrated genomic viewer (IGV) analysis of NRAS exon 5 in parental LLCs compared to 2 LLC NRAS KO subclones (LLC 23 and LLC 46). (**C**) IGV analysis of CMT KRAS G12C subclone 54-10 RNA sequencing data focused on KRAS exon 2, codon 12.

A.

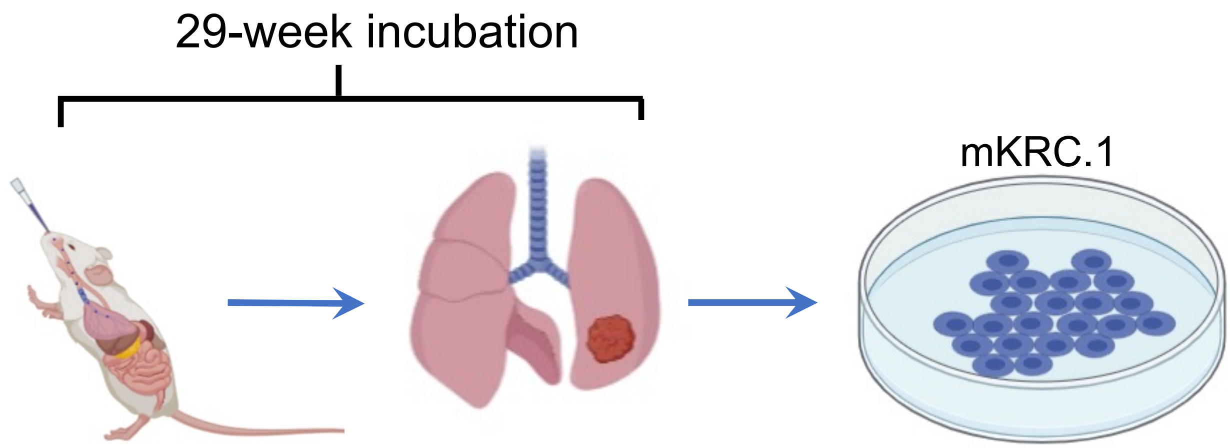

B.

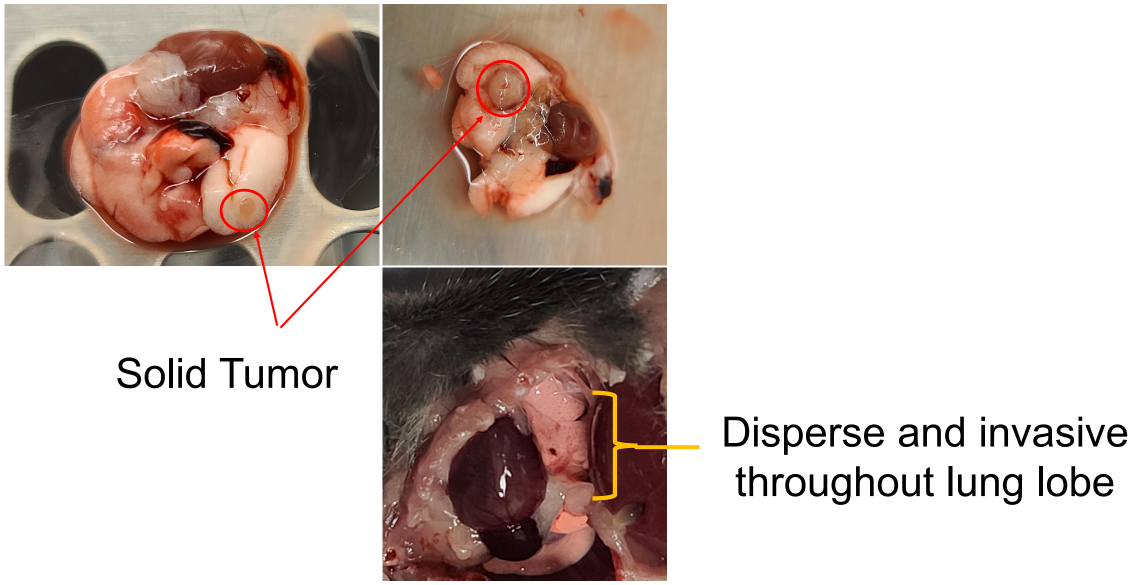

Supplemental Figure S2. Overview of development of mKRC.1 cell line. (**A**) Schematic of intratracheal injections followed by lung resection and cell line development. (**B**) Gross images showing lung tumor nodules and patterns of tumor development within mouse lungs.

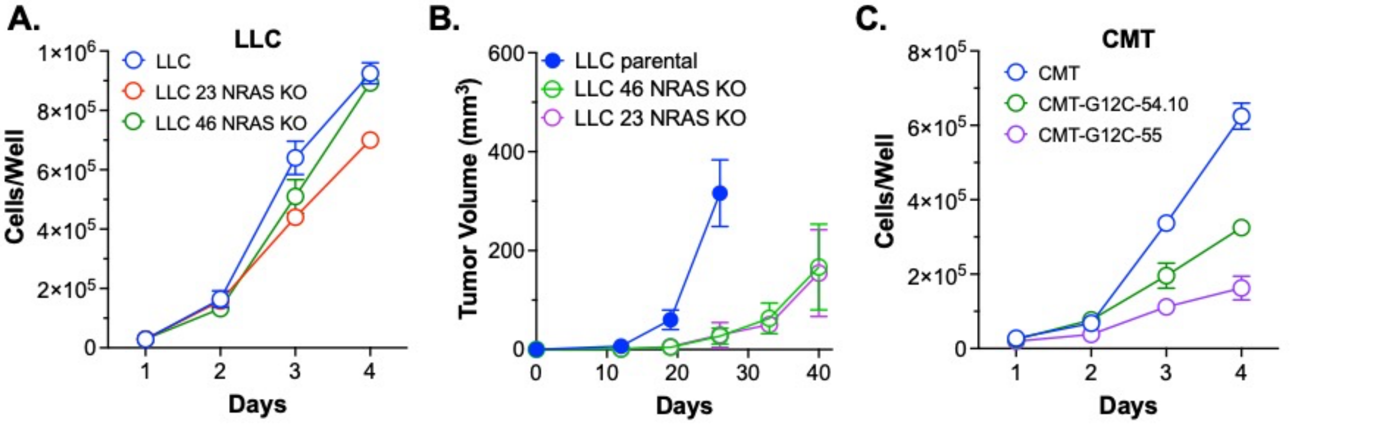

Supplementary Figure S3

Supplementary Figure S3. Baseline growth rates of LLC and CMT167 CRISPR-edited cell lines *in vitro* and *in vivo*. (**A**) and (**C**) Parental and CRISPR-edited cell lines were plated at 25,000 cells per well in 24-well plates and cultured. Duplicate wells were trypsinized and cells counted after 1-4 days. The data are the means and SEM. (**B**) LLC parental, LLC 23 NRAS KO and LLC 46 NRAS KO cell lines were orthotopically implanted into the left lungs of C57BL/6 mice and tumor growth was measured via weekly μCT imaging. The data are the means and SEM of 5 mice per cell line.

A.

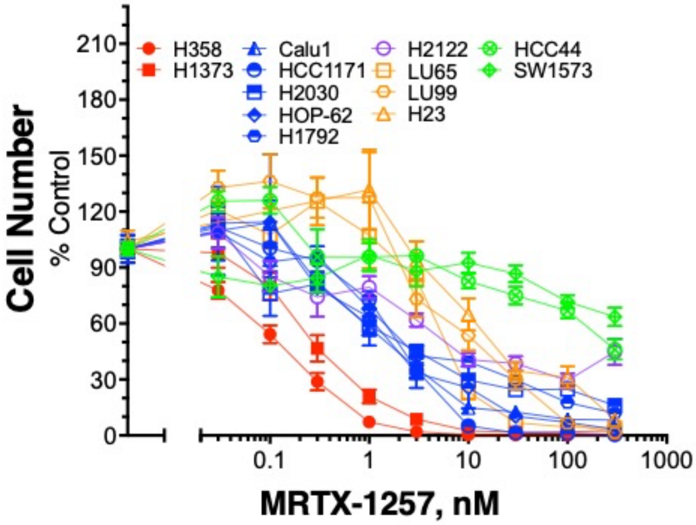

B.

Human KRAS-G12C Lung Cancer Cell lines

	MRTX-1257 IC_{50} (nM)	AMG-510 IC_{50} (nM)
H358	0.11	0.27
H1373	0.30	3.80
Calu1	1.62	2.47
HCC1171	1.96	7.38
H2030	2.69	16.98
HOP-62	1.85	14.7
LU65	6.90	1.96
H1792	3.87	187.2
H2122	7.96	64.8
H23	23.0	92.54
LU99	12.25	N/D
HCC44	185.7	1386
SW1573	355.5	2534

Supplemental Figure S4. Sensitivity of human KRAS-G12C positive lung cancer cell lines to MRTX-1257 and AMG-510. A panel of thirteen human KRAS-G12C mutant lung cancer cell lines were seeded at 100-200 cells/well in 96-well plates and treated for 7-10 days with MRTX-1257 (**A**) or AMG-510 (not shown). Cell number was assayed with CyQUANT reagent. The data are the means and SEM of triplicate measurements presented as percent of the DMSO control treatments. (**B**) Prism 9 was used to calculate the IC_{50} values from the MRTX-1257 and AMG-510 dose-response curves.

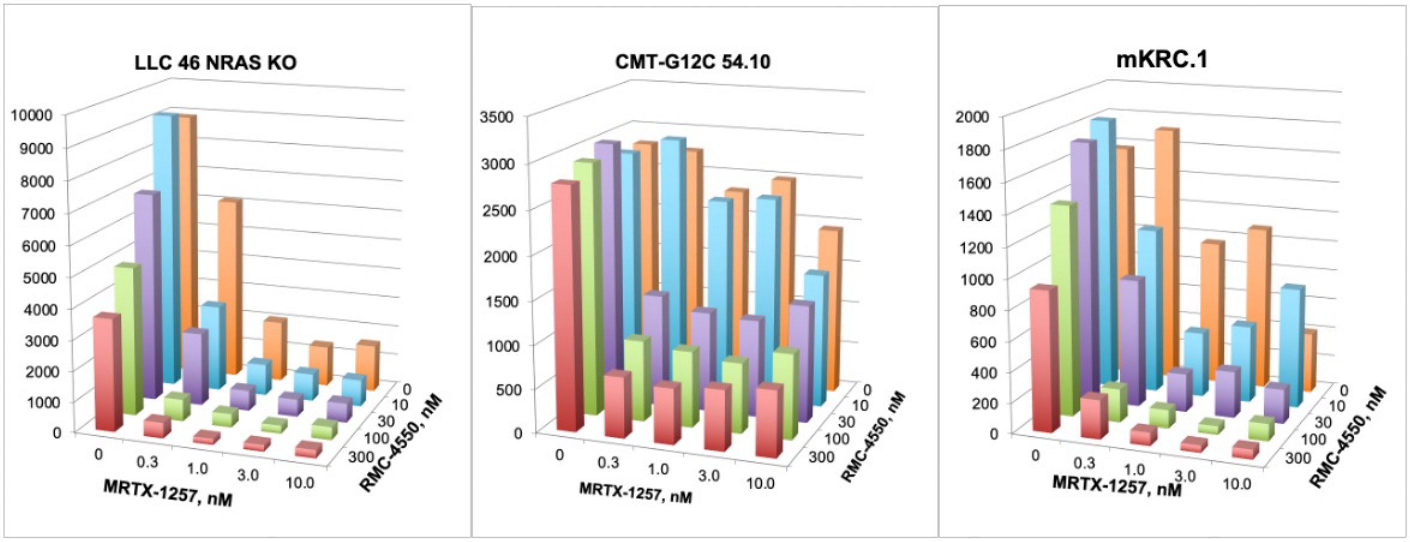

Supplemental Figure S5

Supplemental Figure S5. Primary MRTX-1257-RMC-4550 combination data used for calculating drug synergy. The indicated murine KRAS-G12C cell lines were treated with combinations of MRTX-1257 and RMC-4550 at the concentrations shown in quadruplicate in a 96-well format. After 7-10 days of treatment, cell number was measured with CyQUANT reagent and the average values among the replicates is plotted as shown.

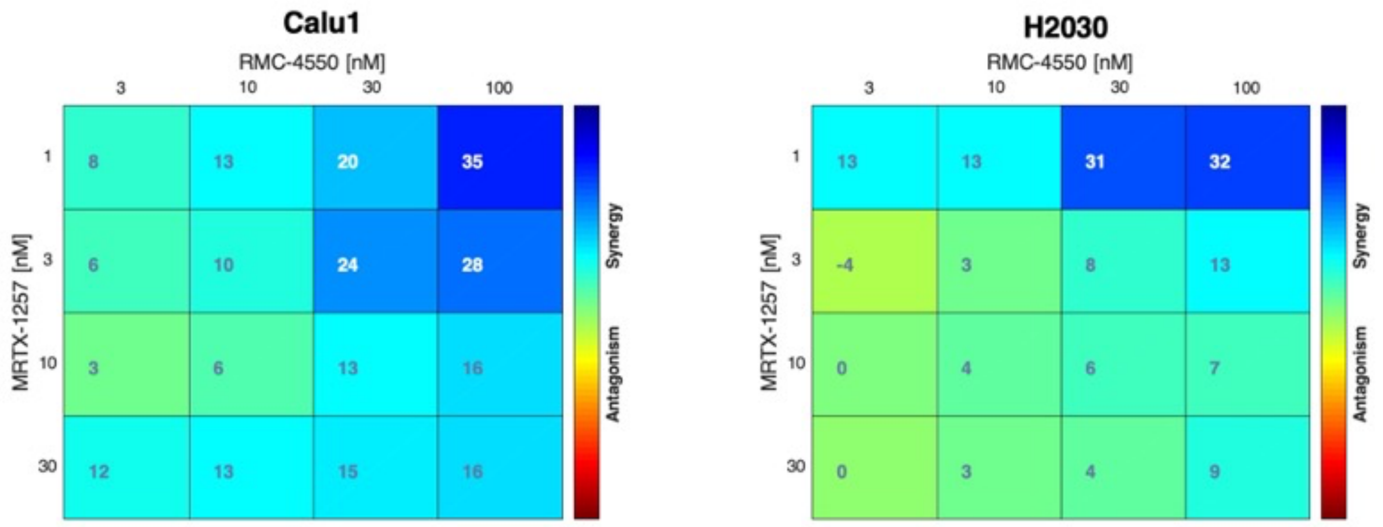

Supplemental Figure S6. Analysis of KRAS^{G12C}-SHP2 inhibitor synergy in human KRAS^{G12C} lung cancer cell lines. Calu1 and H2030 were seeded at 100 cells/well in 96-well plates and treated in duplicate with the indicated concentrations of MRTX-1257 and RMC-4550 for 7-10 days. Cell numbers were assayed with CyQUANT reagent and the resulting data were analyzed with Combenefit for drug synergy using the HSA model.

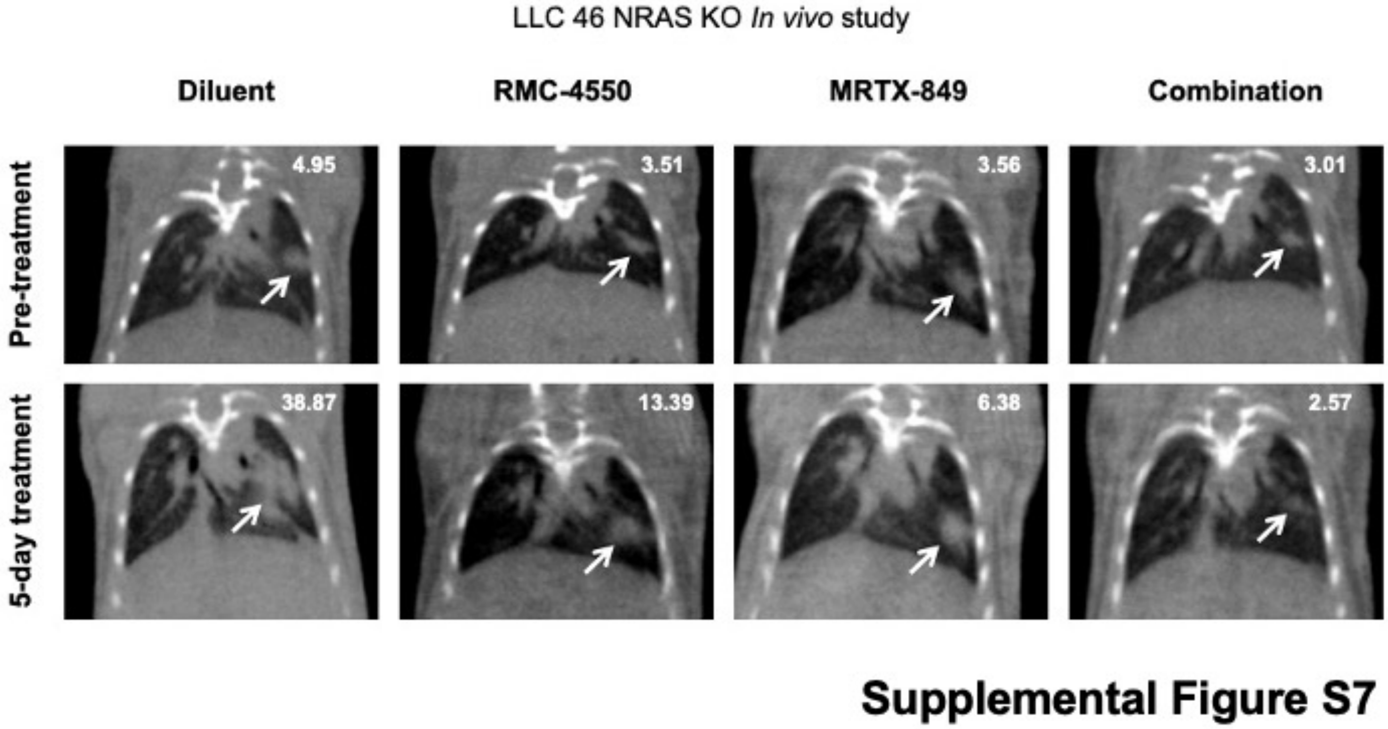

Supplemental Figure S7. MicroCT images from representative orthotopic LLC 46 NRAS KO lung tumors. Representative orthotopic lung tumors from the LLC 46 NRAS KO experiment in Figure 3 are shown from the four experimental groups before and after 5 days of daily treatment. The values in the upper right-hand corner of each image are the tumor volumes calculated by the ITK-SNAP software program.

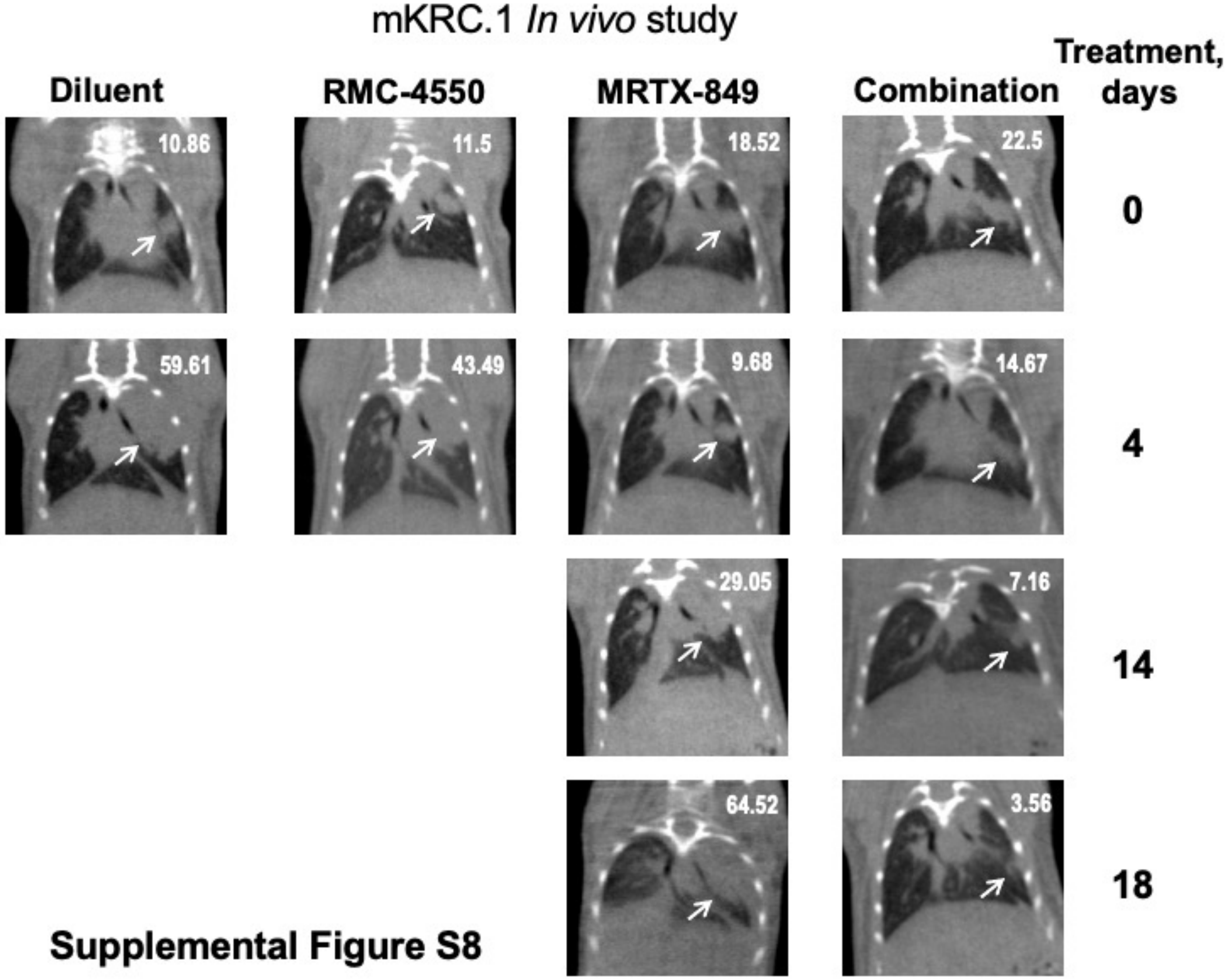

Supplemental Figure S8. MicroCT images from representative orthotopic mKRC.1 lung tumors. Representative orthotopic lung tumors from the mKRC.1 experiment in Figure 4 are shown from the four experimental groups prior to and after the indicated number of days of daily treatment. The values in the upper right-hand corner of each image are the tumor volumes calculated by the ITK-SNAP software program.

Supplementary Table S1. Insertion and deletion mutations detected by variant calling of RNAseq data from CRISPR/Cas9-edited murine KRAS cell lines. Three independent samples from the indicated KRAS-G12C clones as well as parental CMT167 and LLC cells were submitted to RNAseq followed by variant calling as described in the Materials and Methods. Insertion and deletion mutations identified in at least two of the replicate samples and not found to be present in any of the parental cell line replicates are tablulated below.

CMT KRAS-G12C.54.10	CMT KRAS-G12C.55	LLC NRAS-KO.23	LLC NRAS KO.46
Afg3l2:p.K787delinsKKE	9030624J02Rik:p.H704fs	Cherp:p.812_814del	0610010F05Rik:p.V622fs
Brms1:p.E179fs	Afg3l2:p.K787delinsKKE	Gm7244:p.61_61del	1600014C10Rik:p.P82fs
Cwc22:p.R77fs	Amotl1:p.N561fs	Kdm3b:p.E236fs	Anapc2:p.17_19del
D6Ertd527e:p.S374delinsSSS	Atrx:p.V2020fs	Plekhm2:p.508_509del	Apbb1ip:p.149_149del
Dbf4:p.V134fs	B2m:p.T24fs	Pusl1:p.E158fs	Atrx:p.V2020fs
Efcab5:p.Q372fs	Chd1l:p.V624fs	Sgta:p.L247fs	Epha2:p.R891fs
Hgfac:p.N544fs	Dbf4:p.V134fs	Stk11:p.M125fs	Gm7244:p.61_61del
Hnf1b:p.151_154del	Ddx58:p.I460fs	Tpm1:p.K177fs	Il1r1:p.F280fs
Kif12:p.S59delinsSPPGGG	Dynlt3:p.E24fs		Ltbp1:p.Q241fs
Lrba:p.1152_1161del	Eif4g3:p.L983fs		Mink1:p.487_488del
Ltbp3:p.P285fs	Hnf1b:p.151_154del		Nr3c1:p.74_75del
Mars:p.V259fs	Kif12:p.S59delinsSPPGGG		Pex6:p.K49fs
Megf8:p.N2158fs	Lamb3:p.D100fs,		Plekhm2:p.508_509del
Muc4:p.L1347fs	Lig1:p.L689fs		Ppp4c:p.V68fs
Myo6:p.E461fs	Lrba:p.1152_1161del		Prmt1:p.H119fs
Myo9a:p.L2104delinsLL	Msln:p.G30fs		Ptgr2:p.M52fs
Naa16:p.K625fs	Muc4:Np.L1347fs		Pusl1:p.E158fs
Psap:p.Q260fs	Muc4:p.V1684delinsVV		Rnd3:p.N51fs
Rabggta:p.K80fs	Numb:p.T68fs		Rnf10:p.Y623fs
Scnn1a:p.S508fs	Nup153:p.T407fs		Sec24b:p.237_237del
Slc9a5:p.A698fs	Pdlim1:p.P176fs		Tpm1:p.K177fs
Ssfa2p.S166fs	Phkb:p.R619fs		Unc119:p.I204delinsII
Uchl4:p.100_102del	Plxnd1:p.A715fs		Whsc1l1:p.147_147del
Zfp553:p.L401fs	Pold3:p.A219fs		
Zmat3:p.Q90fs	Ppp2r5c:p.I32fs		
	Prmt9:p.S192delinsSI		
	Psap:p.Q260fs		
	Smek2:p.I263fs		
	Ssfa2:p.S166fs		
	Trerf1:p.T1090fs		
	Trim41:p.124_126del		
	Uchl4:p.100_102del		
	Usp16:p.Q392fs		
	Wsb2:p.G351fs		
	Zfp36l1:p.K19fs		
	Zfp553:p.L401fs		

Supplementary Table S2. KRAS-G12C inhibitor IC50 values from dose-response experiments in murine cell lines (Figure 1). Dose-response data were submitted to non-linear fitting using the "log(inhibitor) vs. normalized response" feature in Prism 9. The calculated IC50 values and the 95% confidence intervals (CI) are tablulated. ND, not determined.

	AMG-510		MRTX-1257		MRTX-849		Trametinib	
Cell Line	IC50	95% CI	IC50	95% CI	IC50	95% CI	IC50	95% CI
LLC Parental	236	118 to 530	246	82 to 1035	ND	ND	26	11 to 63
LLC 46 NRAS KO	16.9	10.9 to 26.2	1.6	1.0 to 2.3	44	30 to 64	21	8.0 to 59
LLC 23 NRAS KO	7.4	5.0 to 10.8	2.7	2.0 to 3.7	ND	ND	9.6	5.6 to 16
CMT Parental	Unstable		Unstable		ND	ND	2	1.2 to 3.4
CMT54.10	7.9	5.7 to 11	1.2	0.79 to 1.9	64	30 to 139	0.59	0.44 to 0.78
CMT55	5.1	2.9 to 8.8	0.33	0.14 to 0.77	ND	ND	0.52	0.25 to 1.1
mKRC.1	218.2	99.5 to 565.1	3.1	2.3 to 4.1	5.5	3.6 to 8.3	1.2	0.53 to 2.1
